# Anxiety levels and obsessive compulsion symptoms of pregnant women during the COVID-19 pandemic

**DOI:** 10.4274/tjod.galenos.2020.91455

**Published:** 2020-10-02

**Authors:** Murat Yassa, Ahmet Yassa, Cihangir Yirmibeş, Pınar Birol, Umur Göktuğ Ünlü, Arzu Bilge Tekin, Kemal Sandal, Memiş Ali Mutlu, Gül Çavuşoğlu, Niyazi Tug

**Affiliations:** 1 University of Health Sciences Turkey, Şehit Prof. Dr. İlhan Varank Sancaktepe Training and Research Hospital, Clinic of Obstetrics and Gynecology, İstanbul, Turkey; 2 Tuzla State Hospital, Clinic of Psychiatry, İstanbul, Turkey

**Keywords:** Anxiety, COVID-19, obsessive-compulsion, pregnancy, SARS-CoV-2

## Abstract

**Objective:**

Reliable data regarding maternal mental well-being during the Severe Acute Respiratory syndrome coronavirus-2 (SARS-CoV-2) pandemic are scarce. This study aimed to assess the state/trait anxiety and obsessive-compulsive symptoms of pregnant women and compare those with the non-pregnant population using patient-reported validated outcome measures.

**Materials and Methods:**

This prospective case-control study was conducted at a tertiary ‘Coronavirus Pandemic Hospital’ in İstanbul, Turkey in April, 2020. Pregnant and non-pregnant women were consecutively allocated to two groups regardless of gestational age. The primary outcome was to identify the anxiety levels and obsessive-compulsive symptoms of pregnant women during the SARS-CoV-2 pandemic using the State-Trait Anxiety inventory (STAI) and Maudsley Obsessive-Compulsive inventory (MOCI), respectively.

**Results:**

Two hundred three pregnant women and 101 non-pregnant women were included. The mean STAI-S questionnaire score of pregnant and nonpregnant women was 41.96±9.15 and 46.62±12, respectively (p=0.001). The overall incidence of STAI >40 in pregnant and non-pregnant women was 62.6% and 73.3%, respectively. The mean total score of MOCI was 17.9±6.7 and 15±6.6 in pregnant and non-pregnant women, respectively. The overall incidence of 30-item-MOCI ≥13.1 in pregnant and non-pregnant women was 61.6% (125/203) and 30.7% (31/103), respectively (p<0.001).

**Conclusion:**

State anxiety and obsessive-compulsive symptoms in pregnant women were found increased during the current SARS-CoV-2 pandemic. Pregnant women showed more favourable anxiety levels compared with non-pregnant women. These findings can be used to improve the coping skills of pregnant women during the pandemic, to prepare for the post-pandemic period, and to deal with the long-term mental health impact of COVID-19.

**PRECIS:** Anxiety and obsessive-compulsive symptoms were increased in pregnant women during the SARS-CoV-2 pandemic but anxiety was worse in non-pregnant.

## Introduction

The coronavirus 2019 (COVID-19) pandemic is a global major health crisis that brought with it a profound psychological and psychosocial morbidity^(1)^. The mental health impact of the pandemic may take a long time to be noticed and become completely apparent^(2)^. The future secondary effects of COVID-19 on the general population including the deterioration of financial conditions, quarantine conditions, and psychological reactions during emergencies can cause several negative psychiatric outcomes^(3)^. Those adverse psychological consequences may include depression, stress, anxiety, emotional dysregulation, and may include the exacerbation of pre-existing conditions or symptom^(1,3,4)^.

It is evident that the special populations that are vulnerable to mental health problems will be affected by the secondary psychological impacts of the pandemic to a greater extent^(1)^. Pregnant women, along with children, adolescents, the geriatric population, and patients with pre-existing mental problems deserve special attention.

An important call for action was announced for investigating the psychological effects of the COVID-19 pandemic across the general and vulnerable population, which will be likely required in the event of a second wave of infection^(5)^. Apart from many letters, correspondence, and commentaries, there are only a few large-scale observational studies available investigating the mental consequences of the pandemic to date^(2)^. Observational studies that focused on pregnant women are even more limited^(6,7)^. The knowledge about the short- and long-term maternal mental wellbeing during this pandemic is scant in the literature and thus, potential negative psychological outcomes should be taken as a critical public health problem in order to improve optimal maternity care^(8)^.

A recent review concluded that depressive and anxiety symptoms had been reported in up to 28% of subjects screened for common psychological reactions as a response to the COVID-19 pandemic^(2)^. Concern was also raised about obsessive-compulsive symptoms, which are more common among pregnant women than the general population, are currently being neglected and the possibility of an increase in their frequency and intensity during and after the pandemic^(9,10)^. The number of individuals at risk for obsessive-compulsive disorder (OCD) that would not develop otherwise is thought to have increased along with the fear of pandemic^(11)^.

Given the increased risk for the occurrence of OCD in both the pregnancy and the postpartum period^(12)^, and the increased adverse fetal outcomes related with obsessive-compulsive symptoms during pregnancy such as fetal loss^(13)^, the changes in obsessive-compulsive traits of pregnant women during the current pandemic need to be determined.

An individuals’ response to the pandemic and coping skills are inevitably influenced by multiple psychological and social factors^(1)^. At this point, more objective data are urgently needed to provide reliable detailed information and psychological support to pregnant women^(1,5,14,15)^. In this study, it was aimed to assess the psychological response of pregnant women to the COVID-19 pandemic with a multi-perspective approach. Their state and trait anxiety and obsessive-compulsive traits were evaluated.

## Materials and Methods

This case-control study presents an analysis of prospectively collected data yielded at a single tertiary ‘Coronavirus Pandemic Hospital’ centre in April, 2020. The cohort consisted of a study and a control group from consecutively included women who presented to the outpatient clinic. Women with a confirmed singleton healthy pregnancy were included in the study group regardless of gestational age. The control group comprised non-pregnant women aged between 20 and 50 years. Participants were assigned in a 2:1 fashion. Women either with signs and symptoms of COVID-19 or were suspected of having COVID-19 or diagnosed labour or women with any obstetric indication that required admission to the hospital and women with any current/prior known psychiatric disorder and gynaecological malignancy were excluded from the study prior to enrollment.

Women were asked to complete a form consisting of a demographic enquiry and three validated patient-reported questionnaires to assess the anxiety, obsessive-compulsive symptoms and metacognition, with anonymity preservation. The questionnaires were the State-Trait Anxiety inventory (STAI) and the Maudsley Obsessive-Compulsive inventory (MOCI)^(16,17)^. Demographic data included age, body mass index (BMI), gestational week, parity, economic status, household size, and if the family was responsible for looking after an elder family member. The official minimum wage was used to distinguish low, middle, and high income. The participants completed the questionnaires anonymously, taking an average of 20 minutes to finish. The primary outcome was to identify the anxiety levels and obsessive-compulsive symptoms of pregnant women in the era of current Severe Acute Respiratory syndrome coronavirus-2 (SARS-CoV-2) pandemic. All patients gave written informed consent and approved for publication before data collection. All procedures were in accordance with the 1964 Helsinki Declaration and its later amendments. The Ethics Committee of the University (registry no: 20/203), Scientific Board of the Health Ministry and the local institutional administration board approved the study.

### Measures

### The Spielberger State-Trait Anxiety Inventory

The Spielberger STAI measures the presence and severity of current symptoms of anxiety and a generalized propensity to be anxious^(17)^. The STAI enables to differentiate the temporary condition of state anxiety and the continuity of anxiety, which shows the tendency throughout life by using two separate 20‐item self‐report questionnaires^(18)^. Scoring is reversed for anxiety-absent items. Both subscales have a total score ranging from 20 to 80 points. High scores indicate high levels of anxiety. A cut-off point of 39-40 has been suggested to detect clinically significant symptoms for the S‐Anxiety scale^(17)^.

### MOCI

The MOCI is a 30-item self-report questionnaire using a true/false format, which measures the severity of obsessive-compulsive symptoms and assesses the types of obsessive-compulsive symptoms on four subscales: checking-control, cleaning-hygiene, slowness, and doubting^(19)^. In the 37-item version, seven items were added to the original form to additionally evaluate ruminations^(20)^. Higher scores reflect more severe obsessive-compulsive symptoms. Both scores for 30- and 37-item versions are provided in this study. A reliability generalization meta-analysis found the average mean scores of 11.40 and 13.11 for sub-clinical and clinical samples, respectively^(16)^.

### Statistical Analysis

The SPSS-22 software was used for data analysis. The independent samples t-test was used to compare the means of the scales. Variables are reported by their mean ± standard deviation and mean difference. Internal consistency was assessed using Cronbach’s alpha, and an α value of between 0.70 and 0.95 was considered to reflect good internal consistency. Pearson’s correlation test was used to assess the association between the scales using r-values. A p-value of less than 0.05 was considered significant.

## Results

Two hundred three pregnant women and 101 non-pregnant women were included. The mean age and BMI of the pregnant women were 27.4±5.3 years and 27.83±4.33 kg/m^2^, respectively. The mean age and BMI of the control group were 27.6±4.1 years and 23.48±5.08 kg/m^2^, respectively. The ages of both groups were similar (p=0.698), the BMI was significantly higher in pregnant women with a mean difference of 4.35 kg/m^2^ (p<0.001). Median gestational week of the pregnant women was 35 weeks (interquartile range: 11) and ranged between 4 and 42 weeks. Among all the pregnant women, 8.4% were in the first, 21.7% in the second, and 70% in the third trimester of pregnancies.

Reliability coefficients, mean scores of the primary outcomes, and their comparisons within both groups of anxiety and obsessive-compulsive are presented in Table 1.

**Figure 1:**
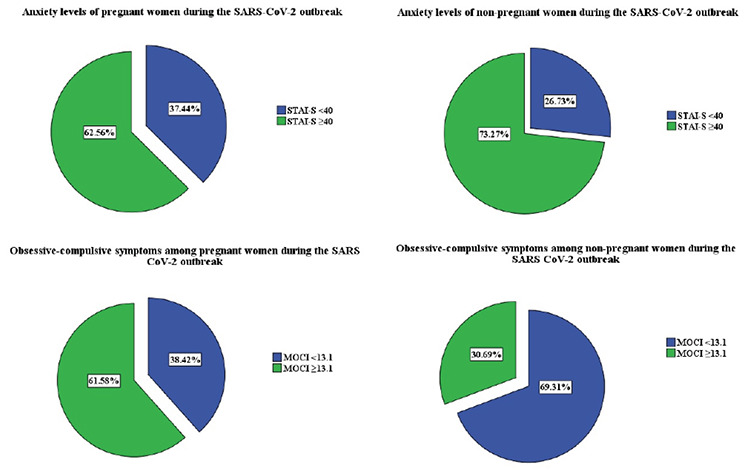
Anxiety levels and obsessive-compulsive symptoms of pregnant and non-pregnant women during the SARS-CoV-2 outbreak The pie chart depicts the proportion of women with unfavourable measure results. Charts at the upper-line show pregnant and nonpregnant women with increased state-anxiety level scores when the cut-off was considered as 40. Charts at the lower line show pregnant and non-pregnant women with increased obsessive-compulsive symptom scores when the cut-off was considered as 13.1. SARS-CoV-2: Severe acute respiratory syndrome coronavirus-2, STAI: State anxiety sub scale of State Trait Anxiety inventory, MOCI: Maudsley Obsessive-compulsive inventory

The mean STAI-S questionnaire score of pregnant and non-pregnant women was 41.96±9.15 and 46.62±12, respectively. The overall incidence of STAI >40 in pregnant and non-pregnant women was 62.6% (76/203) and 73.3% (74/103), respectively (Figure 1). State anxiety levels did not differ among first, second, and third trimesters [p=0.793, f=0.232, One-Way analysis of variance (ANOVA)].

The mean total score of MOCI was 17.9±6.7 and 15±6.6 in pregnant and non-pregnant women, respectively (Table 1). Note, these results belong to the 37-item version of the MOCI. Following re-analysis for the 30-item version to enable future comparisons, the mean total score of the MOCI was found as 15.4±5.1 and 12.4±5.6 in pregnant and non-pregnant women, respectively. The overall incidence of 30-item-MOCI ≥13.1 in pregnant and non-pregnant women was 61.6% (125/203) and 30.7% (31/103), respectively (re-calculated for the first 30 items, Figure 1).

The internal consistency for the total scores of the STAI-S, STAI-T, and MOCI scales was found sufficient at moderate-to-good levels (Table 1). The results of the correlation analyses are presented in Table 2. In the correlation analysis, current anxiety status (STAI-S) was found to be positively associated with all obsessive-compulsion symptoms except the cleaning and hygiene subscale (p<0.01). The associations of state anxiety with other measures were found to be weak to moderate (r<0.60).

The monthly income of the pregnant women was low in 45.8%, medium in 52.2%, and high in 2%. The monthly income of the control group was low in 5.9%, medium in 79.2%, and high in 14.9%. One-Way ANOVA of the measures comparing three different economic status showed a significant difference only in cleaning-hygiene subscale of the metacognition test (p=0.002) and doubting subscale of the MOCI test (p=0.04). An increased cleaning-hygiene score with a mean difference of 1.7 was observed in women with low income compared with those with high income (p=0.004). An increased doubting score with a mean difference of 0.9 was observed in women with low income compared with women with high income (p=0.033).

## Discussion

This survey study showed increased anxiety and obsessive-compulsive symptoms in pregnant women during the current SARS-CoV-2 pandemic. More than 60% of the pregnant women and more than 30% of non-pregnant women reported increased obsessive-compulsive symptoms with regard to the proposed cut-off values^(16)^. Pregnant women showed more favourable anxiety levels compared with non-pregnant women. To the best of our knowledge, this is the first study in the literature to assess the obsessive-compulsive symptoms of pregnant women during the SARS-CoV-2 pandemic and compare those and their anxiety levels in a case-control fashion.

A recent Italian study investigating the psychological impact of COVID-19 in pregnant women found a similar anxiety rate at 68%^(6)^. Another study that validated the STAI in pregnant women addressed an overall score of 35.3 for use in the general pregnant population^(21)^. The mean state anxiety scores of this study were also found considerably higher than those specified levels.

Although both groups expressed higher state anxiety during the pandemic than the normal population in the literature, state anxiety scores were significantly higher in the control group (p=0.001), but similar for trait anxiety (p=0.14). The authors believe that this difference in state anxiety levels between pregnant and non-pregnant women may be related to paid lay-off restrictions specifically for pregnant women and the high self-isolation rates in Turkey. Comparable trait anxiety implies that pregnant women were not significantly more prone to being anxious compared with the control group.

Pregnancy is associated with the onset of obsessive-compulsive symptoms. Forray et al.^(22)^ found that 15% of women who had ever given birth had their obsessive-compulsive disease onset during pregnancy. Brockington et al.^(23)^ observed that one-tenth of pregnant women who were referred to specialist psychiatric services had an obsessive activity or symptoms during pregnancy usually obsessive cleaning or housework, including a new-onset rate of 6%. In this study, pregnant women showed more obsessive traits than non-pregnant women in the MOCI total score (p<0.001) and in the checking, cleaning, and doubting subscales (p<0.005). The mean total scores of MOCI (30-item version, for comparison reasons) were found as 15.4 and 12.4 in pregnant and non-pregnant women, respectively. A reliability generalization meta-analysis found average mean scores of 11.40 for sub-clinical and 13.11 for clinical samples^(16)^. In our cohort, more than half of the pregnant women (61.6%) and almost one-third of the non-pregnant women (30.7%) were found to have higher obsessive symptom scores than the proposed mean values in the literature. More specifically, a mean total score of 7.6 was found in a large cohort of pregnant women who were admitted to the clinic for delivery, which is far below our current findings^(24)^. Although it is known that obsessive-compulsive symptoms generally worsen during pregnancy and puerperium^(25)^, the authors believe that those higher scores than the mean values of the above-mentioned literature should be conveniently linked to the current SARS-CoV-2 pandemic. In this study, current state anxiety status was found to be positively associated with all obsessive-compulsion symptoms except the cleaning and hygiene subscale (p<0.01).

The high obsessive-compulsion symptoms among pregnant women of the current study may be biased due to the lower economic status than that of the non-pregnant women. In this study, 45% of the pregnant women reported having low-income, whereas only 6% of the non-pregnant women had low income. One-quarter of all pregnant women with low socio-economic status seen in primary care settings in Brazil have been shown to have obsessive symptoms^(26)^. However, the significant impact of the COVID-19 pandemic is evident considering the increased rates of obsessive-compulsion symptoms in non-pregnant women when compared with the literature. It has previously been suggested that integrating cognitive behavioural therapy-based prevention programs into antenatal education classes for women at risk of developing OCD was associated with significantly lower levels of obsessive-compulsive traits following childbirth^(27)^. This important finding should be kept in mind during the outbreak, and strategies should be developed to prevent antenatal and postnatal obsessive-compulsive treats. Screening the psychological status of pregnant women may be integrated into universal screening programs^(28)^ to achieve long-term success and reductions in symptoms of anxiety and depression simultaneously in a short time frame.

### Study Limitations

The major limitation of this study is that its design did not include the comparison of psychiatric levels of pregnant women in pre-pandemic versus pandemic period, which would make a clear sense for the effect of the pandemic on pregnant women. However, a strong relationship was found between the target population and the mean scores of MOCI^(16)^; therefore, our results can be used as control scores for further comparisons in future studies. Another strength of this study is the relatively larger and controlled sample size. In addition, this study contributes to the literature by being one of the rare studies that assessed obsessive-compulsive symptoms of pregnant women during the pandemic. The internal consistency of the STAI and MOCI at sufficient levels was also provided in the current study to be used in further studies.

## Conclusion

State anxiety and obsessive-compulsive symptoms in pregnant women were found increased during the current SARS-CoV-2 pandemic. Pregnant women showed increased obsessive-compulsive symptoms and more favourable anxiety compared with non-pregnant women. These findings can be used to improve the coping skills of pregnant women during the pandemic and to deal with the long-term mental health impact of COVID-19.

## Figures and Tables

**Table 1:**
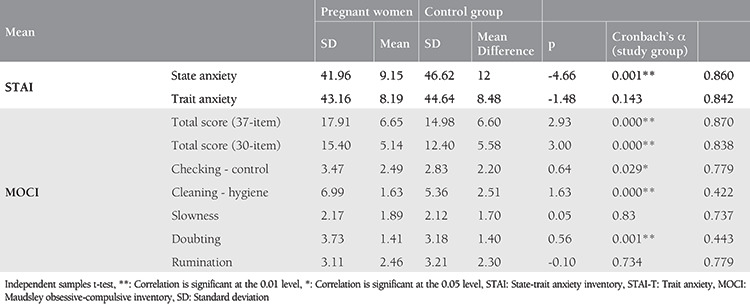
The total and subscale scores of state and trait anxiety and obsessive-compulsive symptoms for pregnant and non-pregnant women

**Table 2:**
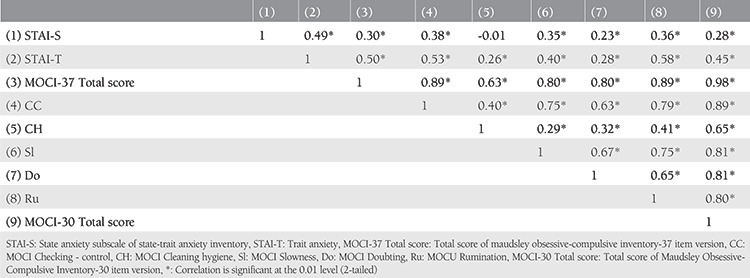
The correlation of objective scales and their subscales
